# Post-Cooking Growth and Survival of *Bacillus cereus* Spores in Rice and Their Enzymatic Activities Leading to Food Spoilage Potential

**DOI:** 10.3390/foods12030626

**Published:** 2023-02-01

**Authors:** Yugenraj Navaneethan, Mohd Esah Effarizah

**Affiliations:** Food Technology Division, School of Industrial Technology, Universiti Sains Malaysia, Penang 11800, Malaysia

**Keywords:** *Bacillus cereus*, rice, microbial enzyme activities

## Abstract

*Bacillus cereus* strains vary in their heat resistance, post-processing survival and growth capacity in foods. Hence, this study was carried out to determine the effect of cooking on the survival and growth of eight *B. cereus* spores in rice at different temperatures in terms of their toxigenic profiles and extracellular enzyme activity. Samples of rice inoculated with different *B. cereus* spores were cooked and stored at 4 °C, 25 °C and 30 °C for up to 7 days, 48 h and 24 h, respectively. Out of eight *B. cereus* strains, four and three spore strains were able to grow at 30 °C and 25 °C post-cooking, respectively. Rapid growth was observed after a minimum of 6 h of incubation at 30 °C. All strains possessed proteolytic activity, whereas lipolytic and amylolytic activities were exhibited by 50% and 12.5% of the strains, respectively. The post-cooking survival and growth capacity of the *B. cereus* strains appeared to be independent of their toxigenic profiles, whereas extracellular enzymatic activities were required for their vegetative growth. Due to the *B. cereus* spores’ abilities to survive cooking and return to their active cellular form, great care should be taken when handling ready-to-eat foods.

## 1. Introduction

*Bacillus cereus* is an important foodborne pathogen with the abilities to contaminate several types of food products at different food production and processing stages and to persist in food due to its remarkable resistance to a wide range of chemical, biological and physical stresses [1,2]. The resistance of *B. cereus* arises from its ability to form endospores, which can remain in a dormant state [3,4]. Consequently, the resistant spores can withstand various processing steps, including heat treatments, which only cause sublethal damage to the spores rather than inactivating them [5,6]. The surviving spores can return to their active cellular form, known as vegetative cells, when the conditions are favorable [7]. This scenario is very much undesirable, as the surviving spores are conducive to food spoilage and food poisoning upon the germination and synthesis of toxins and extracellular enzymes during their vegetative growth [8,9,10].

The heat resistance, germination and growth behaviors of *B. cereus* spores are very intricate and difficult to predict, as these phenotypes differ, even among individual spores of a population, and they also depend on the test media or food models used [3,11,12]. Thus, the complexity of the nature of *B. cereus* spores has always remained a major challenge for the successful eradication of *B. cereus* by food industries, particularly without compromising the quality of end-products [13,14]. 

*B. cereus* is known to cause foodborne illnesses through the production of two types of toxins, which are emetic toxins and enterotoxins [15,16]. The enterotoxins that elicit diarrheal syndromes or toxicoinfections consist of hemolysin BL (Hbl), non-hemolytic enterotoxin (Nhe), cytotoxin K (CytK) and enterotoxin FM (EntFM) [16,17]. Emetic syndrome or food intoxication occurs following the ingestion of an emetic toxin (cereulide) [16]. The illnesses caused by *B. cereus* range from mild, self-limiting symptoms to severe cases, resulting in fatalities [18,19]. 

Apart from playing roles in microbial growth, the production of extracellular enzymes, such as amylase, protease and lipase, contribute to changes in organoleptic properties and the reduction of the shelf life of food products [20,21]. The ropy spoilage in cereals and the bitter flavoring in dairy products due to amylases and proteases, respectively, are some examples of *B. cereus*-enzyme-mediated food spoilage [21,22]. 

Rice (*Oryza sativa*) serves as an important source of nutrition and energy for several billions of people across the globe [23]. Unfortunately, rice and rice-based dishes are often implicated in *B. cereus*-mediated food poisoning incidents [18,24,25,26], indicating that rice is a common vehicle for *B. cereus* outbreaks [15]. The inevitability of the contamination of raw rice by *B. cereus* spores and the violation of time–temperature control and poor hygiene in food preparation and handling are very common, and such practices have resulted in foodborne outbreaks [27,28]. This is because the nutrients, the near-neutral pH and the increased water activity of cooked rice can support *B. cereus* growth and toxin production [29,30]. Hence, a few strategies with respect to time–temperature control have been devised to control the germination and growth of surviving spores from reaching hazardous levels [31,32]. 

This study was conducted to assess the post-cooking survival and growth in rice of eight *B. cereus* spores with different toxigenic profiles in order to provide insights into their heat resistance. The growth of the surviving spores was observed at different temperatures to determine the effects of refrigeration, room temperature and temperature-abused conditions on *B. cereus* survival and growth behavior. The extracellular enzymatic activities of the *B. cereus* strains were determined in order to study whether these characteristics affect the survival and growth of *B. cereus* in cooked rice. This provides insights into the potential risk of food spoilage stemming from the extracellular enzymes produced upon the germination and growth of the surviving spores of *B. cereus*. 

## 2. Materials and Methods

### 2.1. Bacterial Strains

A total of eight strains of *B. cereus* were used in this study. Two of the strains were *B. cereus* DSMZ 4312 (emetic) and ATCC^®^ 14579^TM^ (diarrheal), which were reference strains. The former strain, originally isolated from a patient’s vomit, was obtained from Leibniz Institute, German Collection of Microorganisms and Cell Cultures (Deutsche Sammlung von Mikroorganismen und Zellkulturen) in a freeze-dried form. The latter strain, isolated from air in a cow shed, was obtained in the form of ready-to-use culti-loops (Thermo Scientific, Waltham, MA, USA) derived from the American Type Culture Collection (ATCC). The other six strains, which were previously isolated from ready-to-eat cooked rice [33], were USMBC 01, USMBC 02, USMBC 03, USMBC 04, USMBC 05 and USMBC 06. The eight strains were specifically selected based on their toxigenic traits (the presence of toxin genes) and nutritional characteristics, which had been determined in preliminary studies. This was to ensure that the survivability of the strains of varying toxin gene profiles and nutritional characteristics could be observed. 

The strain USMBC 06 harbored only *ces*, whereas USMBC 01 and USMBC 02 were diarrheagenic strains. The remaining three strains, USMBC 03, USMBC 04 and USMBC 05, were positive for both emetic and diarrheal toxin genes. The toxin gene profiles of each strain are detailed in Table 1. All the isolated and reference strains were maintained in Mixed Colors CryoCare Beads of the Microorganism Preservation System (Scientific Device Laboratory, Des Plaines, IL, USA) at −80 °C. The bacterial cultures were reactivated via inoculation into Tryptic Soy Broth (TSB), followed by incubation at 37 °C for 24 h under shaking (120 rpm), and they were subsequently used as working cultures.

### 2.2. Survival and Outgrowth of Bacillus cereus Spores in Rice after Cooking

#### 2.2.1. Raw Rice Samples

White, polished raw rice was obtained from a local grocery store on Penang Island, Malaysia. The raw rice samples were then UV-sterilized before being used for *B. cereus* inoculation. A proportion of each of the raw rice samples was then stored at room temperature for 24 h before homogenizing and plating on nutrient agar to determine the sterility of the samples. This step was necessary to indicate that the subsequent growth after the cooking of the inoculated raw rice was strictly due to the *B. cereus* inoculation step carried out in the later part of this study. 

#### 2.2.2. Preparation of Spore Suspension 

Unlike the spores, the vegetative cells of *B. cereus* are easily inactivated by thermal processing at high temperatures, such as cooking [14,15]. Hence, the post-cooking survival and growth of *B. cereus* in rice were examined by using *B. cereus* spores. Overnight, *B. cereus* liquid cultures, which were prepared in Section 2.1, were used as working cultures. A volume of 0.1 mL of each *B. cereus* culture was spread onto a sporulation medium containing nutrient agar with the added salts of MnSO_4_ and CaCl_2_ at 0.001% (*w*/*v*). The plates were incubated at 37 °C for 5–6 days. The growth on each plate was suspended in 30 mL cold sterile distilled water and centrifuged six times at 9500× *g* for 8 min. The final pellet was resuspended in an appropriate volume (5 mL) of sterile distilled water and stored at 4 °C [34,35]. Spore enumeration was carried out to determine the concentrations of the prepared spore suspensions.

#### 2.2.3. Post-Cooking Survival and Growth of *Bacillus cereus* in Rice

The survival and growth of the *B. cereus* spores following the cooking of the rice were examined according to the method described by Ankolekar and Labbe [34] with slight modifications. An appropriate volume of the spore suspensions (~10^8^–10^9^) derived from each *B. cereus* strain was inoculated into each raw rice sample of 250 g to give a final concentration of 10^4^ spores/g per rice sample. The inoculated raw rice (250 g) mixed with 600 mL of sterile water was placed in a beaker covered with an aluminum foil, and it was boiled for approximately 15 min until the rice gelatinized. The cooked rice was cooled at room temperature for about 30 min. A 10 g aliquot of the cooked rice sample was incubated at refrigeration temperature (4 °C), room temperature (25 °C) and 30 °C for seven days, 48 h and 24 h, respectively. At an appropriate interval time, the 10 g aliquot was homogenized in 90 mL TSB and plated in duplicate on nutrient agar after serial dilution (up to 10^−4^). The plates were incubated at 37 °C for 24 h to determine the viable count of *B. cereus*. The experimental steps were repeated three times on different days. In each experimental repeat, the above-mentioned steps were also performed by using a negative control (uninoculated rice) to ensure that no cross-contaminations occurred during the incubation or at any point of the experiment. Based on the viable counts, the generation time and the specific growth rate of the *B. cereus* strains were determined by using the following equations:

$$g=\frac{t}{3.3\log\frac{N}{N_{0}}}\;;\;\;\;\mu =\frac{0.693}{g}$$

where *g* = generation time, *N*_0_ = numbers of *B. cereus* at the beginning of the time interval, *N* = numbers of *B. cereus* at the end of the time interval, *t* = time lapse, and *µ* = specific growth rate. 

#### 2.2.4. Determination of Extracellular Enzymatic Activities of *Bacillus cereus*

The production and activity of extracellular amylase, lipase and protease by the eight *B. cereus* strains were determined by culturing the strains on starch, tributyrin with phenol red and skim milk agars, respectively [8,36]. The agars consisted of 1% (*w*/*v*) starch, 1% (*v*/*v*) tributyrin oil and 2% (*w*/*v*) skim milk as substrates. The *B. cereus* strains were spot-inoculated on the appropriate culture media and incubated at 37 °C for 24–48 h. The hydrolysis due to amylase was confirmed by adding iodine to the agars at the end of the incubation, whereas proteolytic and lipolytic activities were determined via direct observations of hydrolysis zones around the bacterial growth. The experiment was repeated three times on different days. 

## 3. Results

### 3.1. Survival and Outgrowth of Bacillus cereus Spores in Rice after Cooking

The spores of four *B. cereus* strains were able to survive the cooking process, and they subsequently germinated into vegetative cells, which were observed in the cooked rice at 30 °C, whereas at 25 °C, the growth of the spores of only three *B. cereus* strains was observed. The detection of the viable counts (surviving spores that germinated and underwent active cellular growth, forming visible colonies) of these *B. cereus* strains following the cooking process indicates their survival and outgrowth capacity. Table 2 shows the viable counts of the *B. cereus* strains observed at different time intervals and incubation temperatures. No viable *B. cereus* was detected in the cooked rice at any incubation time for up to 7 days when the cooked rice was stored at 4 °C. The outgrowth of the germinated spores of the three *B. cereus* strains DSMZ 4312, USMBC 02 and USMBC 04 occurred at both 25 and 30 °C, whereas the viable cell count of the strain USMBC 05 was only observed at 30 °C.

**Figure 1:**
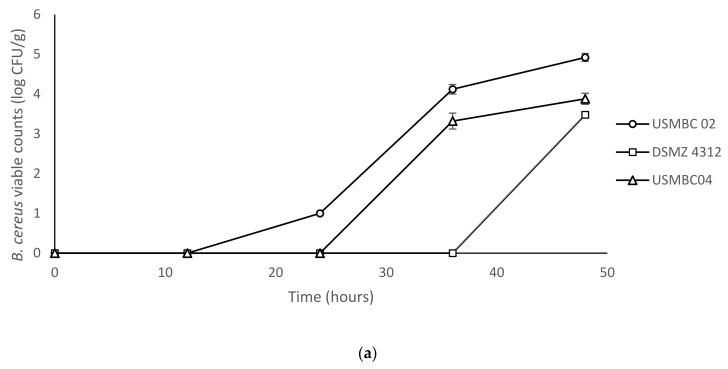

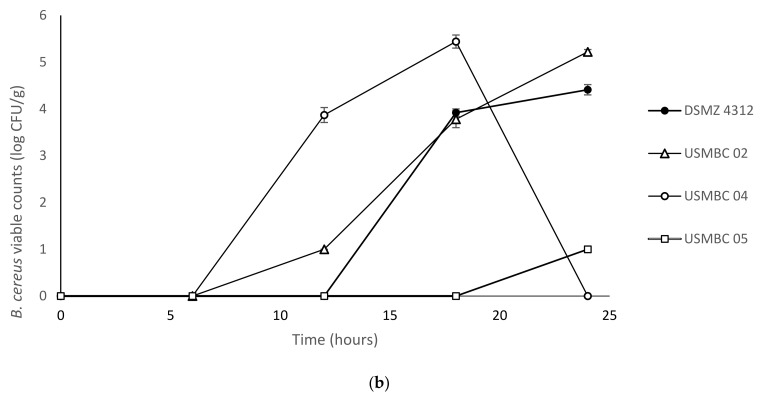
The growth of surviving spores of different *B. cereus* strains in cooked rice stored at (**a**) 25 °C and (**b**) 30 °C. The absence of *B. cereus* colony formation in aerobic plate count is represented by zero log CFU/g (below the limit of detection), whereas 1 log CFU/g indicates below the limit of quantification (LOQ). The values represent the means obtained from three independent experiments ± standard deviations.

In Figure 1a, it can be observed that the growth of the strain USMBC 02 was detected as early as 24 h, whereas the viable counts of USMBC 04 and DSMZ 4312 were first observed after 36 and 48 h of incubation, respectively. The counts of USMBC 02 increased with subsequent incubation times at 25 °C, reaching 4.12 and 4.92 log CFU/g at the 36th and 48th hours, respectively. Out of the three strains, the microbial growth of USMBC 02 was the highest at 25 °C. Lower viable counts of 3.32 and 3.88 log CFU/g were detected by the 36th and 48th hour, respectively, for the strain USMBC 04 in the rice at 25 °C. In comparison to the other two strains, DSMZ 4312 had the lowest count (3.48 log CFU/g) in the cooked rice, and it was only detectable at the end of the incubation at 25 °C. 

Contrarily, four strains (DSMZ 4312, USMBC 02, USMBC 04 and USMBC 05) were able to grow in the rice after the cooking process, with much higher bacterial counts achieved in a shorter incubation time at 30 °C, as shown in Figure 1b. The growth of both USMBC 02 and USMBC 04 was detected by the 12th hour of incubation. The viable count of USMBC 02 by the 12th hour was below the limit of quantification, but USMBC 04 had a substantially higher bacterial count, which was 3.87 log CFU/g. The strain USMBC 04 resumed its growth until the 18th hour, reaching a high bacterial load of more than 5 log CFU/g. By the 24th hour, no viable count of USMBC 04 was observed at 30 °C, but for the strain USMBC 02, the viable count increased from 3.78 log CFU/g to 5.22 log CFU/g during the last 6 h of incubation. The viable count of DSMZ 4312 was first detected after a longer incubation period, that is, 18 hours, with a bacterial count of 3.92 log CFU/g, and it rose to 4.41 log CFU/g by the 24th hour. Unlike the former three strains, the active cellular growth of the heat-injured spores of USMBC 05 was minimal, and it was not detected until the 48th hour of incubation. 

Based on the viable counts of the *B. cereus* strains that exhibited exponential growth during the incubation period, generation time and specific growth rates were determined. Table 3 shows the doubling time and specific growth rates of three *B. cereus* strains at two different temperatures. The growth of *B. cereus* occurred more rapidly at 30 °C, with much higher growth rates (in the range of 0.19–0.60 h^−1^) than at 25 °C for all three strains. The better growth capacity at 30 °C was also exhibited by USMBC 05, which failed to grow at all at lower temperatures. The growth rates of the strains USMBC 02 and USMBC 04 were almost similar at both 25 and 30 °C. Unlike these two strains, no exponential growth was observed for the surviving spores of DSMZ 4312 at 25 °C. Even though the strain DSMZ 4312 grew exponentially from the 18th to the 24th hour at a higher temperature, its growth rate and generation time remained much lower than those of USMBC 02 and USMBC 04. 

### 3.2. Determination of Extracellular Enzymatic Activities of Bacillus cereus 

The amylolytic, proteolytic and lipolytic activities of the *B. cereus* strains were detected qualitatively by using culture media incorporated with appropriate substrates. The breakdown of each substrate results in the formation of an opaque zone around the bacterial growth, indicating degradation by the corresponding enzymes. Table 4 shows the extracellular enzyme activities of the eight *B. cereus* strains. Proteolytic activities on skim milk agar were detected in all eight strains. Half of the strains (ATCC^®^ 14579^TM^, DSMZ 4312, USMBC 01 and USMBC 02) were determined to be lipase-positive. Contrary to the proteolytic and lipolytic activities, extracellular amylase production was not prevalent among the tested strains. Amylolytic activities were devoid in 87.5% of the isolates, and they were only detected in one strain (ATCC^®^ 14579^TM^). The strain ATCC^®^ 14579^TM^ was also the only strain in which the production of all three enzymes was observed.

## 4. Discussion

The ability of dormant *Bacillus cereus* spores to withstand extremely harsh conditions, such as those encountered in natural environments and food processing steps, is remarkable. This ability has, in turn, become a challenge pertaining to food safety issues. The results obtained in this study can be linked to two major aspects of spore-forming organism: (1) the heat resistance of the spores subjected to cooking and (2) the germination efficiency, outgrowth capacity and proliferation of the heat-injured, surviving spores in rice. In the context of heat resistance, the spores of four *B. cereus* strains (DSMZ 4312, USMBC 02, USMBC 04 and USMBC 05) can be stated to be resistant to normal rice cooking temperatures, as the viable counts of these spores were still present after cooking (boiling for 15 min). A higher heat resistance is more commonly observed in emetic *B. cereus* than its diarrheagenic counterparts, thus leading to the frequent association of emetic properties with elevated heat tolerance [12,37]. In accordance, in this study, the spores of three (DSMZ 4312, USMBC 04 and USMBC 05) out the four strains that were not inactivated were positive for the emetic toxin gene (*ces*). 

Nevertheless, no viable counts were detected for the spores of the two *ces*-positive strains, USMBC 03 and USMBC 06, whereas the successful resumption of vegetative growth by the diarrheagenic spores of USMBC 02 (devoid of *ces*) following cooking was observed. This indicates that the heat resistance of *B. cereus* is independent of its toxigenic properties. In comparison to emetic strains, certain diarrheal strains of *B. cereus* with either higher or similar (under similar testing conditions) heat resistances have been reported [12,37]. The various amounts of specific nutrients in different types of foods may create a microenvironment, providing spores with a competitive advantage and protection against harsh conditions, or the nutrients could possibly play significant roles in the activation of the genes required for spore resistance and repair mechanisms. Based on the exponential increase in the *B. cereus* cells of three strains (DSMZ 4312, USMBC 02 and USMBC 04) upon survival of the cooking process, it is clear that these *B. cereus* strains are well-equipped to survive and thrive in rice in spite of their different toxigenic profiles. All these findings provide substantial evidence corroborating that the heat resistance of *B. cereus*, which is a key determining factor of its survival during thermal processing, cannot be predicted on the basis of its toxigenic properties. 

Apart from heat resistance, the post-cooking growth of the *B. cereus* in the rice in this study can also be explained in terms of the germination, outgrowth and proliferation (vegetative growth) of the strains. The superior growth capacities of the surviving spores of all four strains at 30 °C compared to those at lower temperatures point towards the mesophilic nature of these strains. Similar to this study, increased growth rates of *B. cereus* at higher temperatures (30–35 °C) compared to at room temperature (25 °C) have been observed in different food models, such as spinach and UHT milk [38,39]. The growth rate of DSMZ 4312 being lower than that of USMBC 02 and USMBC 04 indicates the superdormant nature of *B. cereus* DSMZ 4312 spores. A superdormant (SD) nature is characterized by a greater propensity of spores to retain dormancy due to limited numbers of germinant receptors, resulting in subpopulations of spores that either do not undergo germination or that germinate very slowly, even in the presence of sufficient germination-triggering agents [9,40]. 

In addition to the inherent properties of the surviving spores, spore recovery is also largely dependent on the recovery media or food matrices. Earlier studies have reported that the recovery and germination of *B. cereus* spores occurred less efficiently on rice media despite a considerably shorter duration of heat application (45–60 s) [6], as compared to about 15 min in this study. The longer heat treatment in the present study was necessary to mimic a domestic cooking procedure, which requires rice gelatinization to occur. Regardless of the thermal treatment duration, heat-treated spores tend to exhibit relatively much more delayed growth in order to undergo spore repair, and different media or food matrices possess varying efficiencies to support spore repair and recovery processes [6]. The efficiency of recovery media depends on the presence of specific nutrients that trigger spore germination, and they are equally important for metabolic activities during vegetative growth [7,9]. Since the microbial utilization of energy (nutrient) sources is largely aided by hydrolytic enzymes [41], the extracellular enzyme activities of the eight *B. cereus* strains were deemed to be increasingly relevant. 

Milled white rice generally has carbohydrate, protein and lipid contents (g per 100 g rice) of 77–78, 6.3–7.1 and 0.3–0.5, respectively [23]. Hence, the amylolytic, proteolytic and lipolytic activities of the *B. cereus* strains were detected. Following germination, the growth and proliferation of the surviving *B. cereus* spores appeared to be dependent on their enzymatic activities. The viable counts of DSMZ 4312 and USMBC 02 continued to increase at 30 °C, reaching 4.41 and 5.22 log CFU/g, respectively, whereas the growth of USMBC 04 reached a decline phase following its exponential growth from the 12th to the 18th hour. The strain USMBC 04 depended mainly on protein metabolism (proteolysis) for sustenance, and upon the deprivation of metabolizable nutrients after its exponential growth, the active cellular growth of USMBC 04 was arrested. Contrary to the strain USMBC 04, the strains DSMZ 4312 and USMBC 02, which exhibited both proteolytic and lipolytic activities, were able to sustain growth at 30 °C until the end of the incubation period. Upon the depletion of proteins, the lipolytic activities of these strains possibly enabled the utilization of the fats present to support their vegetative mode of growth for an extended period. This strongly suggests that extracellular enzymatic activities play significant roles in maintaining the active vegetative growth of *B. cereus*. The influence of amylase on the post-cooking growth behavior of *B. cereus* in the rice was unable to be studied, as the sole amylase-producing strain used in the present study, ATCC^®^ 14579^TM^, did not grow after the cooking process. As vegetative cells and germinated spores are more prone to inactivation by stresses, such as heat, than dormant spores [9], the spores of ATCC^®^ 14579^TM^ might have lost dormancy upon sensing metabolizable nutrients (carbohydrates in rice) in abundance, and they were much more easily inactivated during rice cooking than the amylase-negative strains. This indicates that the abilities to retain spore dormancy and its associated resistance phenotypes are also closely intertwined with the nutritional characteristics of spore formers. The nutritional characteristics of *B. cereus* strains also give rise to food spoilage risk stemming from the hydrolytic actions of their extracellular enzymes [4,21]. 

This study’s results also demonstrate that domestic cooking steps are insufficient to kill *B. cereus* spores, even when the contamination level is 10^4^ CFU/g. Interestingly, the surviving spores could germinate and grow, reaching much higher counts than the initial inoculum level. In natural settings, the spore contamination could be several folds higher. The ineffectiveness of food processing steps to eradicate *B. cereus* spores underlines the necessity to control contamination, specifically starting at earlier stages of the food supply chain to reduce the risk in the end-products. 

The strains USMBC 01, USMBC 03, USMBC 05 and USMBC 06, which were either unable to grow or exhibited limited growth in the rice following cooking, were originally isolated from ready-to-eat (RTE) cooked rice. Since the spores of these strains possess poor surviving capacity when subjected to cooking, their occurrence in RTE cooked rice, as detected in an earlier study [33], highlights their potential as post-cooking contaminants. As for surviving spores, the absence of their growth for up to 12 and 6 h at 25 and 30 °C, respectively, indicates that cooked rice is only safe for consumption within a certain duration, depending on the storage conditions, and this further corroborates the critical role of time–temperature control in hazard analysis and critical control point (HACCP) system. The failure of surviving *B. cereus* spores to germinate and grow at 4 °C for as long as 7 days shows that proper refrigeration is required until the point of consumption or serving. Hence, great caution and care in the handling of RTE cooked foods are of paramount importance not only to manage the risks associated with surviving, persistent spores but also to avoid post-processing contamination due to reasons such as bad hygiene. 

Upon time–temperature abuse, the surviving spores of DSMZ 4312, USMBC 02 and USMBC 04, which had high bacterial counts, are likely to produce toxins due to the presence of virulence genes. It is noteworthy that *B. cereus* DSMZ 4312 is a reference cereulide-producing strain isolated from an emetic outbreak [42], and, thus, the survival of DSMZ 4312 spores is highly likely to result in food intoxication. As food intoxication involves cereulide production in foods [10], it is crucial to avoid subjecting foods contaminated with cereulide-producing *B. cereus* to temperatures conducive for the pathogen’s growth and toxin synthesis. Otherwise, subsequent reheating steps will not inactivate the pre-synthesized toxin [25] due to the highly stable nature of cereulide [43]. The growth of the spores of USMBC 02 and USMBC04 in rice after the cooking process indicates the potential risks of diarrheal disease development, as they harbor enterotoxin-associated genes (positive for *nhe*, *cytK* and *entFM*).

## 5. Conclusions

The results of this study confirm that the domestic cooking process of rice is insufficient to inactivate the spores of B. *cereus*. The growth of the surviving spores was observed after a minimum of 6 h of incubation at 25–30 °C. This indicates that cooked foods should be consumed immediately or rapidly cooled to 4 °C when not consumed within 6 h, as per the FDA Food Code guidelines, to ensure that the germination and growth of *B. cereus* can be prevented from reaching potentially hazardous levels. Hence, thorough and appropriate steps during food handling with respect to time–temperature control are proven to be critical to protect against the hazards pertaining to this pathogen. The ability of *B. cereus* to survive the cooking process and its subsequent growth are independent of its toxigenic properties. The presence and actions of the extracellular enzymes of *B. cereus* are crucial to support the vegetative growth of this organism. 

## Figures and Tables

**Table 1:** Toxigenic profiles of the *B. cereus* strains used in this study.

Strains	Toxin Genes Profiles
ATCC^®^ 14579^TM^	*hblACD*, *nheABC*, *cytK*, *entFM*
DSMZ 4312	*ces*
USMBC 01	*nheABC*, *cytK*, *entFM*
USMBC 02	*nheBC*, *cytK*, *entFM*
USMBC 03	*hblCD*, *nheABC*, *cytK*, *entFM*, *ces*
USMBC 04	*nheBC*, *cytK*, *entFM*, *ces*
USMBC 05	*hblCD*, *nheBC*, *cytK*, *entFM*, *ces*
USMBC 06	*ces*

**Table 2:** The viable counts of *B. cereus* spores after cooking at different storage temperatures.

Strain	Temperatures (°C)	Time Interval	Mean log CFU/g ± SD
DSMZ 4312	4	Up to 7 days	ND
	25	Up to 36 h	ND
		48 h	3.48 ± 0.05
	30	Up to 12 h	ND
		18 h	3.92 ± 0.08
		24 h	4.41 ± 0.11
ATCC^®^ 14579^TM^	4	Up to 7 days	ND
	25	Up to 48 h	
	30	Up to 24 h	
USMBC 01	4	Up to 7 days	ND
	25	Up to 48 h	
	30	Up to 24 h	
USMBC 02	4	Up to 7 days	ND
	25	12 h	ND
		24 h	LOQ
		36 h	4.12 ± 0.12
		48 h	4.92 ± 0.10
	30	6 h	ND
		12 h	LOQ
		18 h	3.78 ± 0.18
		24 h	5.22 ± 0.05
USMBC 03	4	Up to 7 days	ND
	25	Up to 48 h	ND
	30	Up to 24 h	ND
USMBC 04	4	Up to 7 days	ND
	25	Up to 24 h	ND
		36 h	3.32 ± 0.20
		48 h	3.88 ± 0.14
	30	6 h	ND
		12 h	3.87 ± 0.16
		18 h	5.44 ± 0.14
		24 h	ND
USMBC 05	4	Up to 7 days	ND
	25	Up to 48 h	ND
	30	Up to 18 h	ND
		24 h	LOQ
USMBC 06	4	Up to 7 days	ND
	25	Up to 48 h	ND
	30	Up to 24 h	ND

‘ND’ (not detected) indicates below the limit of detection (<10 CFU/g); ‘LOQ’ indicates number of colonies below the limit of quantification (less than 25 CFU).

**Table 3:** Generation time and specific growth rate of *B. cereus* strains at 25 °C and 30 °C.

Strain	25 °C		30 °C	
	Generation Time (h)	SGR (h^−1^)	Generation Time (h)	SGR (h^−1^)
DSMZ 4312	ND	ND	3.72	0.19
USMBC 02	4.55	0.15	1.26	0.55
USMBC 04	6.49	0.11	1.16	0.60

ND, not determined. SGR, specific growth rate.

**Table 4:** Nutritional characteristics of *B. cereus* strains.

	Amylase	Protease	Lipase
ATCC^®^ 14579^TM^	+	+	+
DSMZ 4312	−	+	+
USMBC 01	−	+	+
USMBC 02	−	+	+
USMBC 03	−	+	−
USMBC 04	−	+	−
USMBC 05	−	+	−
USMBC 06	−	+	−

‘+’ indicates the presence of enzyme activity; ‘−’ indicates no enzyme activity observed.

## Data Availability

Data supporting the findings of this research are available from the corresponding author or co-author upon reasonable request.
